# HIF-1****α**** had Pivotal Effects on Downregulation of miR-210 Decreasing Viability and Inducing Apoptosis in Hypoxic Chondrocytes

**DOI:** 10.1155/2014/876363

**Published:** 2014-03-25

**Authors:** Zhiqiang Chang, Lifeng Huo, Yimin Wu, Pei Zhang

**Affiliations:** ^1^Surgical Department of Cervical Spine, The Second Affiliated Hospital of Inner Mongolia Medical University, 1 Yingfang Dao Hohehot, Mongolia 010030, China; ^2^Medical School of Nantong University, 19 Qixiu Road, Nantong, Jiangsu 226001, China

## Abstract

Hypoxia-inducible factor 1-alpha (HIF-1**α**) and some microRNA (miRNAs) play pivotal roles in response to hypoxia-related physiologic and pathophysiologic responses. Up to date, the regulatory mechanisms of these molecules were largely unknown in chondrocytes. In this study, to study the mechanisms of degradation and homeostasis of chondrocytes, the effects of miRNAs and HIF-1**α** on chondrocytes in physiologic environment were investigated. We found that the overexpression of miR-210 and HIF-1**α** was present on hypoxia in C28/I2 human chondrocytes significantly by qRT-PCR and western plot. Further study displayed that miR-210 played positive role as a promoter in regulation and its regulated molecules (bcl-xl and PHD-2) in C28/I2 cells on hypoxia by silenced miR-210, silenced HIF-1**α**, and adding miR-210. Moreover, downregulated miR-210 could significantly repress the viability and increase the apoptosis in C28/I2 cells on hypoxia, compared to those on normoxia. Furthermore, miR-210 could not modulate viability and apoptosis in C28/I2 cells with the HIF-1**α** knockdown on hypoxia and normoxia. Taken together, this study demonstrated that the MiR-210 was involved in an HIF-1**α**-dependent way in C28/I2 human chondrocytes for the first time. It also suggested that miR-210 downregulation decreased viability and induced apoptosis in hypoxic chondrocytes depending on HIF-1**α**.

## 1. Introduction

Oxygen is essential to life for all higher organisms. Because of the absence of vasculature, intervertebral disc and articular cartilage play physiological functions and are present upon hypoxic environment in the whole life [1]. Chondrocytes, the resident cells of cartilage and intervertebral disc, are a vital part in human, as they play important roles in articulation of joints and also homeostasis of joint and intervertebral disc [2]. It is highly associated with abnormality of chondrocytes that cervical spondylosis such as cervical osteoarthritis, myelopathy, and radiculopathy caused by degeneration of the intervertebral disc and osteoarthritis characterized by the degradation of articular cartilage [2–4].

Hypoxia-inducible factor 1-alpha (HIF-1*α*), subunit of HIF-1, has pivotal effects on cellular response to hypoxia. HIF-1*α* can modulate lots of genes, which subsequently regulate the pathologic processes, including carcinogenesis, immunity, angiogenesis, proliferation, and apoptosis [5–7]. In addition, in skeletal development and cartilage, HIF-1*α* plays multifunctional roles [1, 8]. The expression of HIF-1*α* was detectable in normal chondrocytes or osteoarthritic chondrocytes under hypoxic condition, and the HIF-1*α* is higher in osteoarthritic chondrocytes than that in normal chondrocytes [9]. Inflammatory factor such as TNF*α* can stimulate the HIF-1*α*, which sustains ATP levels and matrix synthesis in chondrocytes adaptive to the inflammation [9–11]. So it is concluded that HIF-1*α* is a necessary factor in chondrocytes response to hypoxia on the one hand, and on the other hand, the HIF-1*α* may be stimulated by inflammatory factors and take essential functions in response to the inflammation in chondrocytes [12]. The necessary effects of HIF-1*α* on cartilage or chondrocytes are verified clearly, yet the understanding of HIF-1*α* in humans is still limited.

MicroRNAs (miRNAs), composed of about 22 nucleotides, regulate about 60% gene expression by targeting the 3′-untranslated regions (3′-UTRs) to result in negative modulation of relevant mRNAs for expression [13]. miRNAs take significant effect on cellular biology and pathophysiologic regulatory pathways [14, 15]. In chondrocytes, miR-140 is documented abundantly owing to its functions of specific to cartilaginous tissues in zebrafish and mouse [16, 17]. Moreover, miR-140 null rats are deformity according to defects in growth plate cartilage of long bones [18]. Besides the research on miR-140, there are lots of dysregulated miRNAs to be discovered in primary chondrocytes (differentiated versus dedifferentiated cells or normal versus osteoarthritic chondrocytes), such as miR-675 and miR-145 [19–21]. In terms of interactions of miRNA and HIF-1*α*, more reports refer to its interactions in cancer rather than in chondrocytes, which are exposed to hypoxia in the physiological environment and in which the necessary roles of HIF-1*α* on hypoxia are taken. So the reports on interactions of miRNAs and HIF-1*α* in chondrocytes is important and need to be understood.

In this research, we focus on expression of miR-210 and HIF-1*α* on hypoxic environment, the relationships of miR-210 and HIF-1*α*, and the possible regulatory mechanisms of miR-210 in hypoxic chondrocytes.

## 2. Materials and Methods

### 2.1. Cell Culture

C28/I2 human chondrocytes were acquired from the Type Culture Collection of the Chinese Academy of Sciences. C28/I2 cells were grown in Dulbecco's modified Eagle's medium (DMEM) containing 10% FBS and antibiotics (50 units/mL penicillin and 50 *μ*g/mL streptomycin; Invitrogen) for 12 h, 24 h, 48 h, and 72 h. Cells were treated with hypoxia in Anoxomat chambers (Mart Microbiology, Lichtenvoorde, The Netherlands) for physiological hypoxia (5% O_2_) or normoxia (21% O_2_) at 37°C.

### 2.2. RNA Extraction and Measurements of miRNAs and mRNA

RNeasy kits (Qiagen, Valencia, CA, USA) were performed to extract total mRNA from cells. miRNAs was extracted by mirVANA miRNA isolation kit (Ambion, Austin, TX, USA). The mirVana qRT-PCR miRNA Detection Kit (Ambion, Austin, TX, USA) and qRT-PCR Primer Sets were utilized to detect the expression of miR-210. qRT-PCR (quantitative real-time PCR) was performed on ABI 7500 with SYBR green PCR kits (Applied Biosystems). Data were normalized by using 2^−ΔΔCt^ method as relative quantification. The results of miRNAs or mRNA by qRT-PCR were normalized to U6 RNA or GAPDH expression level, respectively. The primers were used as follows. HIF-1*α*: F-ATC GCG GGG ACC GAT T and R-CGA CGT TCA GAA CTT ATC TTT TTC TT. PHD-2: F-ACC ATG AAC AAG CAC GGC ATC TGC and R-GAC GTC TTT GCT GACTGA ATT GGG CTT. BCL-xL: F-CTG TGC GTG GAA AGC GTA G and R-CTC GGC TGC TGC ATT GTT C. GAPDH: F-GCA CCG TCA AGG CTG AGA AC and R-ATG GTG GTG AAG ACG CCA GT.

### 2.3. Transient Transfection

The miR-210 mimics and anti-miR of miR-210 were purchased from Ambion (Ambion, Austin, TX, USA). The antimiR negative control and control RNA (miR-210 mimics control) (Ambion, Austin, TX, USA) were used as its endogenous reference. HIF-1*α* was silenced by using small interfering RNA (siRNAs; Dharmacon Inc.) and nonespecific siRNA was used as a negative control. C28/I2 cells were transfected with miR-210 mimics (20 nM) or anti-miRs (50 nM) or siRNAs of HIF-1*α* (30 nM) by siPORT NeoFX Transfection Agent (Ambion, Austin, TX, USA) as manufacturer's protocol. Cells were harvested after transfection as indicated time on normoxia or hypoxia.

### 2.4. Western Blotting

Western blotting was performed using standard procedure as reference [22]. Briefly C28/I2 chondrocytes were lysed and proteins were fractionated by 10% SDSPAGE gels. Proteins were electrotransferred onto nitrocellulose membranes (Millipore). Immunoblots were performed by primary antibodies. Then, the ECL detection systems (Super Signal West Femto, Pierce) were utilized to capture the signals after incubation with horseradish-peroxidase-conjugated secondary antibodies (Pierce). The following primary antibodies were used: anti-HIF-1*α* (BD Biosciences Inc., Franklin Lakes, NJ, USA) and anti-*β*-actin (Sigma, St. Louis, MO, USA).

### 2.5. MTT Assay

Cell viability was assayed by a 3-(4,5-dimethylthiazol-2-yl)-2, 5-diphenyl tetrazolium bromide (MTT) assay (Sigma, St. Louis, MO, USA) as indicated time on normoxia or hypoxia after being treated with indicated experiments. In this assay, MTT was added to the cells at 37°C for 4 h. After incubation, MTT-containing medium was discarded and dimethyl sulfoxide (DMSO) was performed to dissolve formazan crystals. Optical densities (OD) were measured at 490 nm by Versamax microplate reader (Molecular Devices, Sunnyvale, CA, USA). Viability was normalized by OD value/cell number and 48 h normoxic culture treated with control RNA and si-control was denoted as 100%.

### 2.6. Apoptosis Analysis

Cells apoptosis analysis was treated by annexin V-FITC apoptosis detection kit (Sigma-Aldrich, St. Louis, MI, USA). Briefly, cells were harvested after indicated experiments and cell suspensions were fixed overnight with ice-cold 70% ethanol. Then, cells were stained with propidium iodide or annexin V-FITC after centrifugation and resuspensions. Apoptotic cells were conducted according to the annexin-V-fluorescein isothiocyanate (FITC) manufacturer instructions (KeyGen Biotech. Nanjing, China). Analyses were performed by a flow cytometer (BD FACScan). The results were expressed as the percentage of apoptotic cells from the total cells.

### 2.7. Statistical Analysis

Independent experiments were performed in triplicate or more than triplicate as indicated in figure legends. All data were expressed as mean ± standard deviation (SD). The SPSS 18.0 was used for general statistical analysis. Comparison among multiple samples was made by ANOVA. Student's *t*-test was used to compare two groups. *P* < 0.05 was considered to be statistically significant difference.

## 3. Results

### 3.1. Hypoxia Induced the Up-Steam HIF-1*α* and miR-210 in C28/I2 Cells

**Figure 1:**
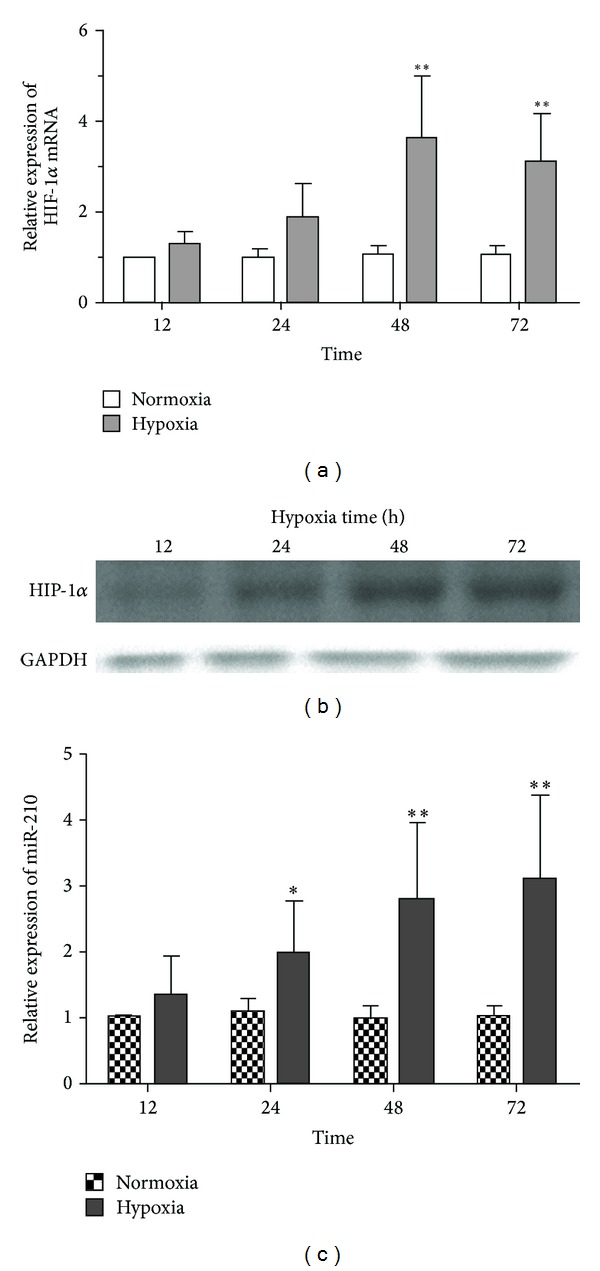
The expression of miR-210 and HIF-1*α* was assayed by qRT-PCR and western plot on hypoxia and normoxia in C28/I2 human chondrocytes. C28/I2 cells were expose to hypoxia or normoxia at 12 h, 24 h, 48 h, and 72 h. Then, the HIF-1*α* mRNA was detected by qRT-PCR in cells (a). The expression of miR-210 on hypoxia was contrasted with that on normoxia (c). Expression of HIF-1*α* protein was also confirmed by western plot at 12 h, 24 h, 48 h, and 72 h in hypoxic C28/I2 cells (b). Five individual experiments were conducted. Student's *t*-test was performed to statistically analyzed. The significant differences were denoted as *P < 0.05 and **P < 0.01.

To show the hypoxia responsiveness of chondrocytes, we analyzed the expression of HIF-1*α* and miR-210 in C28/I2 cells on hypoxic exposure, compared with that on normoxia. Experiments were conducted to expose the cells to 5% oxygen (hypoxia) or 21% oxygen (normoxia) at 12 h, 24 h, 48 h, and 72 h. The relative expression of HIF-1*α* mRNA was displayed in Figure 1(a). The results showed the HIF-1*α* mRNA was increased significantly with time going on and increased HIF-1*α* mRNA reached the plateau on hypoxia at 48 h (*P* = 0.003). The protein expression of HIF-1*α* was undetectable upon normoxic situation (data not showed) and the HIF-1*α* protein upon hypoxia was tested by western plot as shown in Figure 1(b). With the overexpression of HIF-1*α*, time course of miR-210 expression was assessed on hypoxic or normoxic exposure also, as shown in Figure 1(c). It displayed that the expression of miR-210 was enhanced significantly on hypoxic exposure, compared with that on normoxic exposure. The maximum differences in miR-210 expression took place at 72 h in C28/I2 cells (*P* = 0.007). All the findings illustrated that hypoxia induced the upregulated HIF-1*α* and miR-210 expression in C28/I2 cells. It also was supposed that the upregulated HIF-1*α* might be associated with overexpression of miR-210 in C28/I2 cells.

### 3.2. MiR-210 Was Involved in an HIF-1*α*-Dependent Way in C28/I2 Cells

**Figure 2:**
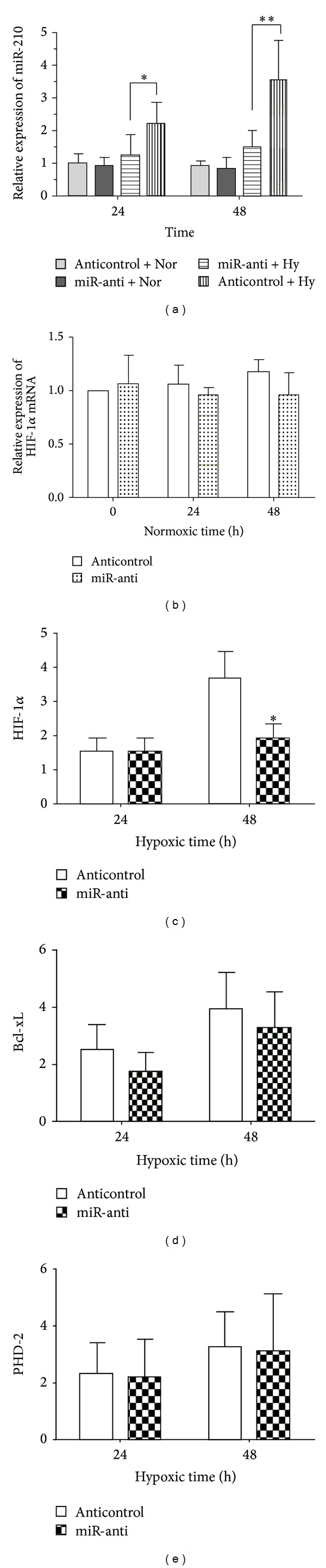
Downregulated miR-210 affected the expression of HIF-1*α* and HIF-1*α*-regulated molecules (BCL-xl and PHD-2) in C28/I2 cells on hypoxia and normoxia. Cells was harvested at 24 h and 48 h on hypoxia or on normoxia after being transfected with miR-anti or anticontrol. Then, the qRT-PCR assay was conducted to examine the expression of HIF-1*α* (b) on normoxia. The same method was utilized to analyze the differences in expression of HIF-1*α* (c), BCL-xl (d), and PHD-2 (e) mRNA between in cells transfected with miR-anti and that with anticontrol on hypoxia. The expression of miR-210 was also confirmed in these groups (a). The qRT-PCR was performed in three individual experiments. Significant differences were denoted as *P < 0.05 and **P < 0.01.

To further investigate the relationship of miR-210 and HIF-1*α*, we assessed the expression of HIF-1*α* according to repressed miR-210 by transfection with miR-anti in C28/I2 cells on normoxic or hypoxic exposure to 24 h and 48 h. Firstly, the relative expression of miR-210 was detected by qRT-PCR to verify the efficiency of transfection. We found the significantly decreased miR-210 in cells treated with miR-anti on hypoxia, as shown in Figure 2(a). Then, there was no change in the relative expression of HIF-1*α* mRNA between cells with miR-anti and cells with anticontrol on normoxia (Figure 2(b)). Moreover, we discovered the downexpression of HIF-1*α* mRNA under hypoxia situation at 48 h in cells transfected with miR-anti significantly, compared to those with anticontrol (Figure 2(c)). Notwithstanding there was no statistically significant reduction in HIF-1*α* regulated molecules Bcl-xl, and PHD-2 in C28/I2 cells on hypoxia, it was obvious the mean level of expression of Bcl-xl, and PHD-2 in cells with miR-anti was repressed slighter than that with anticontrol, especially the expression of Bcl-xl (Figures 2(d) and 2(e)). All these data revealed that the artificial downexpression of miR-210 could depressed the expression of HIF-1*α* and HIF-1*α* associated molecules under hypoxic situation.

**Figure 3:**
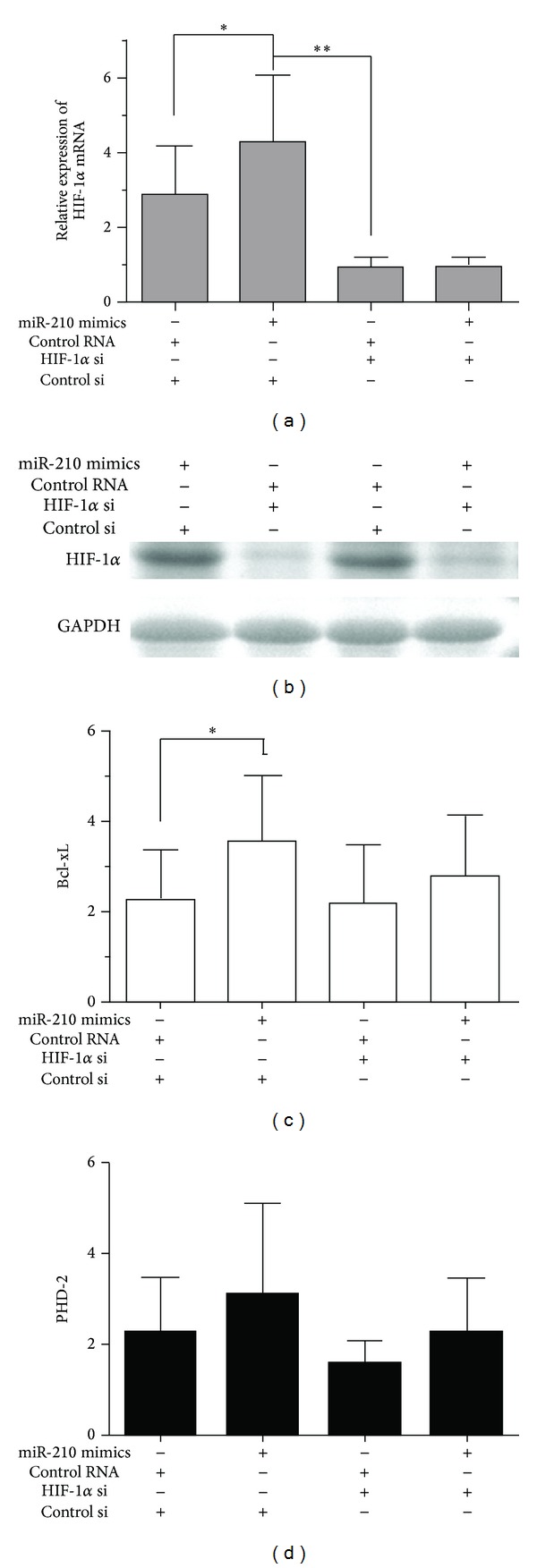
Overexpression of miR-210 increased expression of HIF-1*α* and HIF-1*α*-regulated molecules (BCL-xl and PHD-2) in hypoxic C28/I2 cells. The expression of HIF-1*α* (a), Bcl-xL (c), and PHD-2 (d) was examined by qRT-PCR after 48 h transfection of HIF-1*α* si or miR-210 mimics in hypoxic C28/I2 cells. The knockdown of HIF-1*α* was verified by western plot in the four groups (b). The experiments were performed in triplicate separately. Comparison was made by ANOVA (*P < 0.05 and **P < 0.01).

In addition, we evaluated the effect of miR-210 on HIF-1*α* in C28/I2 cells by knocking down the HIF-1*α* and artificially adding miR-210 upon hypoxic exposure to 48 h. There was dramatically decreased expression of HIF-1*α* as well as HIF-1*α* associated molecules in cells with silencing HIF-1*α* genes compared to those with si-control. Artificially adding miR-210 could remarkably affect the expression of HIF-1*α* in C28/I2 cells rather than that in cells with silenced HIF-1*α* genes. Nevertheless, the expression of Bcl-xl and PHD-2 mRNA was significantly increased either in cells with knockdown HIF-1*α* or in cells without gene knockdown by artificially adding miR-210 (Figure 3). It was suggested that Bcl-xl and PHD-2 were modulated by multimolecules, besides mainly by HIF-1*α*. All these data suggested that miR-210 was involved in an HIF-1*α*-dependent way in C28/I2 cells.

### 3.3. Decreased miR-210 Repressed the Cell Viability and Proliferation in Chondrocytes

**Figure 4:**
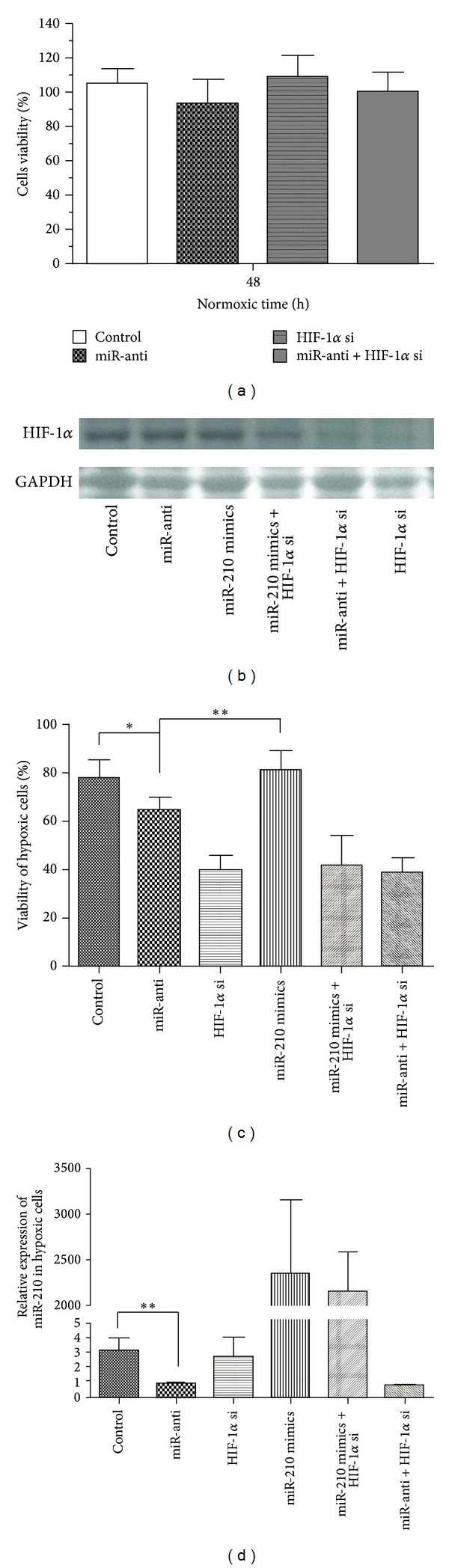
Downregulated miR-210 decreased viability in hypoxic C28/I2 cells depending on HIF-1*α*. Cell viability was detected after 48 culture by MTT assay after being treated with miR-210 mimics, miR-anti, or HIF-1*α* si on normoxia or hypoxia. Data of three individual experiments was normalized and statistically analyzed by ANOVA. (a) Cell viability on normoxia. (b) Western plot assay of HIF-1*α* protein in hypoxic cells treated with miR-210 mimics, miR-anti, or HIF-1*α* si. (c) Cell viability on hypoxia (*P < 0.05, **P < 0.01). (d) Expression of miR-210 in hypoxic cells treated with miR-210 mimics, miR-anti, or HIF-1*α* si (**P < 0.01).

Cell viability and proliferation were examined in hypoxic or normoxic C28/I2 cells by MTT assay at 48 h. The results showed that there was no obvious change in viability of normoxic C28/I2 cells, in which the expression of miR-210 or/and the HIF-1*α* was knocked down (Figure 4(a)). It demonstrated the miR-210 and HIF-1*α* had no effect on viability of chondrocytes upon normoxic exposure, whereas downregulation of miR-210 could significantly decrease the cell viability upon hypoxic situation, in contrast to that with no silenced miR-210 in nonsilenced HIF-1*α* C28/I2 cells. What is more, the hypoxic cell viability was remarkably lower in silenced HIF-1*α* cells than the others (Figure 4(c)). We also detect the expression of HIF-1*α* (Figure 4(b)) and miR-210 (Figure 4(d)) to confirm the efficiency of gene knockdown at the same time. All data illustrated that the HIF-1*α* played a centraxonial role in miR-210 modulating the viability of hypoxic C28/I2 cells; on the other hand, downregulated miR-210 decreased the cell viability depending in HIF-1*α*-dependent way.

### 3.4. Decreased miR-210 Induced Hypoxic Chondrocytes Apoptosis

**Figure 5:**
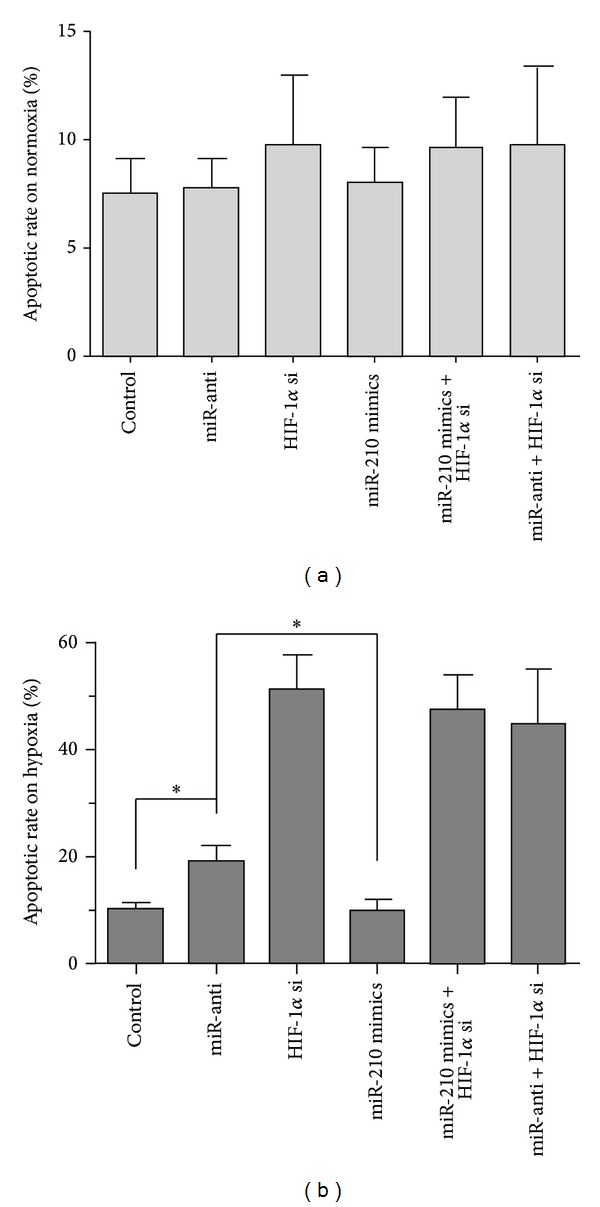
Downexpression of miR-210 increased apoptosis via HIF-1*α* in hypoxic C28/I2 cells. Apoptosis of C28/I2 cells transfected with miR-210 mimics, miR-anti, or HIF-1*α* si was measured by flow cytometry after 48 h upon hypoxia or normoxia. (a) Apoptotic rate in C28/I2 cells on normoxia. (b) Apoptotic rate in hypoxic C28/I2 cells. Three individual experiments were conducted. The significant differences were denoted as *P < 0.05.

Chondrocytes apoptosis was investigated by TUNEL and flow cytometry upon hypoxic or normoxic situation at 48 h. There was no significant difference in apoptosis of normoxic C28/I2 cells, while the C28/I2 cells were treated with miR-anti, miR-210 mimics, or HIF-1*α* si. It was suggested that miR-210 and HIF-1*α* could not affect apoptosis of the C28/I2 cells upon normoxia (Figure 5(a)). To further observe the influences of miR-210 on the hypoxic chondrocytes, we treated cells on hypoxia with the same method as those on normoxia. It was found that downregulated miR-210 stimulated the apoptosis compared to that with control RNA and miR-210 mimics in nonsilenced HIF-1*α* cells. Furthermore, HIF-1*α* knockdown affected the apoptosis significantly, and adding miR-210 mimics did not change the effect on apoptosis which happened in HIF-1*α* silenced cells (Figure 5(b)). All the results revealed that the downstream miR-210 could not affect the apoptosis in normoxic C28/I2 cells, and downstream miR-210 enhanced the apoptosis of cells upon hypoxic situation depending on the HIF-1*α*.

## 4. Discussion

Chondrocytes, the resident cells of cartilage and intervertebral disc, have crucial effects on articulation of joints and homeostasis of joint and intervertebral disc upon hypoxia in the whole life [1, 2]. HIF-1*α*, a key regulator of the transcriptional response to hypoxia, plays important roles in normal chondrocytes or osteoarthritic chondrocytes [7, 9]. miRNAs take specific functions on cartilaginous tissues also [16–18]. Lots of reports are documented on the interaction of miRNAs and HIF-1*α* in cancer cells and epithelial cells [23, 24]. However, there is no report on interaction of miRNAs and HIF-1*α* in chondrocytes.

In this research, to study the life cycle of chondrocytes in physiological environment, we compared the expression of miR-210 and HIF-1*α* in C28/I2 cells upon hypoxia with that upon normoxia. Interestingly, we discovered the overexpression of miR-210 and HIF-1*α* upon hypoxia simultaneously, as shown in Figure 1. It is declared that hypoxia might induce the overexpression of HIF-1*α* as well as the upregulated miR-210 in C28/I2 cells.

It is known that HIF-1*α* plays a key role in hypoxia-related physiologic and pathophysiologic responses and there are lots of miRNAs modulated by hypoxia to be identified [25, 26]. It is verified that miRNAs and HIF-1*α* modulate each other directly or indirectly upon hypoxia since there are the links between oxygen-specific stress factors and gene expression control [24, 27, 28]. In miR-210 respect, miR-210 is modulated by HIF-1*α* according to directly binding to HRE (hypoxia responsive element), which is the proximal miR-210 promoter [29]. miR-210 abundance was enhanced in HIF-1*α*-dependent manner following parasite infection in human primary macrophages [30]. Moreover, miR-210 is a hypoxia-inducible factor (HIF)-1 target gene in response to hypoxia in various cancer cell lines [31–33]. It has been demonstrated MiR-210, one of specific sets of microRNA molecules induced by hypoxia, is robustly upregulated by HIF-1*α*. However, no report refers to hypoxia inducing the activation of the HIF-1*α*/miR-210 signaling pathway in chondrocytes. Previously, we found the upregulated HIF-1*α* and miR-210 in C28/I2 cells. It was assumed that HIF-1*α*/miR-210 signaling pathway might be activated by hypoxia in C28/I2 cells. The overexpression of miR-210 was added artificially and the genes of HIF-1*α* and miR-210 were silenced in C28/I2 cells to confirm the hypothesis. The results illustrated that hypoxic regulation of miR-210 is HIF-1*α* independent in C28/I2 cells, as shown in Figures 2 and 3. Up to date, it is the first report on hypoxia inducing the activation of the HIF-1*α*/miR-210 signaling pathway in chondrocytes.

The effects of HIF-1*α* on diverse physiological processes such as cell proliferation, metabolism, apoptosis, and angiogenesis were mainly mediated by its targets, including phosphoglycerate kinase, prolyhydroxylases (PHD), BCL-xL, and VEGF [34–36]. The physiological effect of HIF-1*α* which was modulated by miR-210 was indicated through detecting the targets of HIF-1*α*, for example, PHD and BCL-xL. So we also reflected the effect of HIF-1*α* regulation by miR-210 according to assessing the downsteam factors (PHD and BCL-xL). It was shown that the effects of miR-210 on Bcl-xL and PHD-2 were coincident with those on HIF-1*α*. These results demonstrated that MiR-210 was involved in a HIF-1*α*-dependent way in C28/I2 cells also.

miR-210 had been conformed as protective to transformed cells, endothelial cells, and mesenchymal stem cells in prohibiting apoptosis in response to hypoxia [37–39]. In the present research, we also found that the downregulated miR-210 significantly increased the apoptotic rate of chondrocytes in response to hypoxia (Figure 5(b)). It was documented that repressed miR-210 decreased the proliferation in carcinoma of the head and neck as well as adenocarcinoma of the pancreas [40, 41]. Conversely, Zhang et al. had revealed that overexpression of this miRNA appeared to decrease proliferation in transformed cells [42]. So it is uncertain if miR-210 acts as promoter or inhibitor in proliferation. In our study, we discovered that the miR-210 played a role as a positive regulator of viability in hypoxic chondrocytes (Figure 4(c)). Furthermore, we detect the apoptotic rate and viability in silenced HIF-1*α* hypoxia chondrocytes with downregulated miR-210 (Figures 4(c) and 5(b)). The results showed that the sharply enhanced apoptotic rate and depressed viability took place in silenced HIF-1*α* hypoxia chondrocytes. These findings revealed that the effects of HIF-1*α* on response to hypoxia were pivotal in chondrocytes and also demonstrated that the downregulated miR-210 induced the apoptosis and decreased the viability in hypoxic chondrocytes in an HIF-1*α*-dependent way.

## 5. Conclusions 

We firstly reported the hypoxia inducing the activation of the HIF-1*α*/miR-210 signaling pathway in chondrocytes. We also revealed that the depressed miR-210 modulated the enhanced apoptosis and the repressed viability in hypoxic chondrocytes in an HIF-1*α*-dependent way. However, the confirmation of direct targets of miR-210 which have effect on response to hypoxia is necessary and will be challenging in chondrocytes.
